# Oxymatrine inhibits non–small cell lung cancer via suppression of EGFR signaling pathway

**DOI:** 10.1002/cam4.1269

**Published:** 2017-12-13

**Authors:** Wei Li, Xinfang Yu, Shiming Tan, Wenbin Liu, Li Zhou, Haidan Liu

**Affiliations:** ^1^ Department of Cardiovascular Surgery The Second Xiangya Hospital of Central South University Changsha Hunan 410011 China; ^2^ Clinical Center for Gene Diagnosis and Therapy The Second Xiangya Hospital of Central South University Changsha Hunan 410011 China; ^3^ Department of Radiology The Third Xiangya Hospital of Central South University Changsha Hunan 410013 China; ^4^ Cell Transplantation and Gene Therapy Institute The Third Xiangya Hospital of Central South University Changsha Hunan 410013 China; ^5^ Department of Cancer Biology Lerner Research Institute Cleveland Clinic 9500 Euclid Avenue Cleveland Ohio 44195 USA; ^6^ Department of Hemopathology The Third Xiangya Hospital of Central South University Changsha Hunan 410013 China; ^7^ Department of Pathology Hunan Cancer Hospital The Affiliated Cancer Hospital of Xiangya School of Medicine Central South University Changsha Hunan 410013 China; ^8^ Department of Pathology Xiangya Hospital of Central South University Changsha Hunan 410008 China

**Keywords:** Akt, cyclin D1, epidermal growth factor receptor, non–small cell lung cancer, oxymatrine

## Abstract

Epidermal growth factor receptor (EGFR) plays a crucial role in human non–small cell lung cancer (NSCLC) tumorigenesis. In this study, oxymatrine was identified as an EGFR signaling pathway inhibitor in NSCLC. Oxymatrine inhibited anchorage‐dependent and independent growth of NSCLC cell lines but had no cytotoxicity in normal lung cells. We found that exposure to oxymatrine not only suppressed the activity of wild‐type EGFR but also inhibited the activation of exon 19 deletion and L858R/T790M mutated EGFR. Flow cytometry analysis suggested that oxymatrine‐induced cell cycle G0/G1 arrest was dependent on EGFR‐Akt signaling. Exogenous overexpression of Myr‐Akt rescued cyclin D1 expression in HCC827 cells. Moreover, oxymatrine prominently suppressed tumor growth in a xenograft mouse model. Thus, oxymatrine appears to be a novel therapeutic agent for NSCLC treatment.

## Introduction

Non–small cell lung cancer (NSCLC) is one of the most lethal cancers worldwide for both men and women 1. Although the early diagnosis was improved and the standard treatment was developed during the past few decades, NSCLC is often diagnosed at an advanced stage, and the overall 5‐year survival rate remains less than 15% 2, 3, 4. Numerous evidence revealed that host genetic susceptibility is closely linked to increased NSCLC risk 5. Overexpressed or hyperactivated of epidermal growth factor receptor (EGFR) is always detected in NSCLC. Recently, The EGFR targeting therapy has played a central role in advanced NSCLC treatment. Novel targeted therapeutic agents, including tyrosine kinase inhibitors (TKIs), such as gefitinib, erlotinib, and osimertinib, or monoclonal antibody cetuximab, have been developed to interfere with EGFR signaling, and show promise in the treatment of advanced NSCLC 6, 7, 8, 9, 10.

Oxymatrine, one of the major alkaloid components found in the roots of Sophora species, have various pharmacological activities and are demonstrated to have anti‐inflammatory, antiallergic, antivirus, antifibrotic, and cardiovascular protective effects 11. Recent studies have demonstrated that oxymatrine has anticancer potentials in various human cancer cell lines, including breast cancer 12, gastric cancer 13, bladder cancer 14, hepatocellular carcinoma 15, colon cancer 16, ovarian cancer 17, and prostate cancer 18. The molecular mechanism studies have demonstrated that induction of cell cycle arrest 19, promotion of apoptosis 14, inhibition of angiogenesis 20 and suppression of metastasis 17 were involved in oxymatrine‐mediated antitumor effect. Nonetheless, the antitumor activity of oxymatrine in NSCLC, as well as its potential target was not clear.

In this study, the efficacy of oxymatrine against NSCLC was evaluated *in vitro* and *in vivo*. We discovered firstly that oxymatrine inhibited the activation of both wild‐type and mutant EGFR. Our data revealed that oxymatrine‐induced cell cycle arrest was mainly dependent on the suppression effect of EGFR‐Akt‐cyclin D1 signaling pathway.

## Materials and Methods

### Reagents and antibodies

Chemicals, including NaCl, Tris, SDS, and oxymatrine (>98%) were purchased from Sigma (St. Louis, MO). LY294002, PD98059, and Erlotinib were purchased from Selleck Chemicals (Houston, TX). The RPMI‐1640 and fetal bovine serum (FBS) for cell culture were from Invitrogen (Grand Island, NY). Antibodies against p‐EGFR (Tyr1068), EGFR, p‐ERK1/2 (Thr202/Tyr204), p‐Akt (Ser473), Akt1, Akt, ERK1/2, cyclin D1, cyclin A, cyclin E, CDK4, p21, CDK2, and p27 were obtained from Cell Signaling Technology, Inc. (Beverly, MA). Antibody against *β*‐actin was from Sigma. Anti‐Ki67 was from Abcam (Cambridge, MA).

### Cell culture and transfection

Cells, including A549, H1975, HCC827, HBE, MRC5, and NL20 from American Type Culture Collection (ATCC, Manassas, VA) were cultured at 37°C in a humidified incubator with 5% CO_2_. A549, H1975, and HCC827 were cultured in RPMI‐1640 medium supplemented with 1% antibiotics and 10% FBS. The MRC5 lung fibroblast cell was cultured in Eagle's Minimum Essential Medium supplemented with 1% antibiotics and 10% FBS. The HBE bronchus epithelial cell was grown in keratinocyte‐serum‐free medium with 0.05 mg/mL bovine pituitary extract, 5 ng/mL human recombinant EGF, 0.005 mg/mL insulin and supplemented with 500 ng/mL hydrocortisone. The NL20 bronchus epithelial cell was cultured in Ham's F12 medium with 2.7 g/L glucose, 1.5 g/L sodium bicarbonate, 2.0 mmol/L l‐glutamine, 0.005 mg/mL insulin, 0.1 mmol/L nonessential amino acids, 0.001 mg/mL transferrin, 10 ng/mL epidermal growth factor, 500 ng/mL hydrocortisone and supplemented with 4% FBS. The jetPEI (Qbiogene, Inc., Montreal, Canada) transfection reagent was used for transfection experiments. The cell lysate was extracted for Western blot analysis.

### Immunoblotting

Nonidet P‐40 buffer (50 mmol/L Tris‐Cl, pH 8.0, 150 mmol/L NaCl, 0.5% Nonidet P‐40, and protease inhibitor mixture) was used for protein extraction. After protein concentration, the denatured proteins (30 *μ*g) were subject to sodium‐dodecyl‐sulfate–polyacrylamide gel electrophoresis and incubated with primary antibodies and an alkaline phosphatase (AP)‐conjugated secondary antibody. Proteins were visualized by chemiluminescence (Amersham Biosciences, Piscataway, NJ).

### MTS assay

The cells were counted and seeded (2 × 10^3^) into 96‐well plates and incubated with oxymatrine as indicated. Cell proliferation was assessed by MTS assay (Promega, Madison, WI) according to the manufacturer's protocol.

### Anchorage‐independent growth

The anchorage‐independent growth assay was conducted as described previously 21. Briefly, NSCLC cells were suspended (8000 cells/mL) in 1 mL of Eagle's basal medium containing 0.3% agar, 1% antibiotics, 10% FBS, and different concentrations of oxymatrine. The mixture was then overlaid into six‐well plates containing a 0.6% agar base and different concentrations of oxymatrine. The cells were maintained in an incubator for 1–2 weeks, the colonies were counted using the Image‐Pro Plus software program (Media Cybernetics, Silver Spring, MD).

### Flow cytometry

Flow cytometry assay was performed as described previously 22. Briefly, the cells were treated with oxymatrine and suspended at a concentration of 1 × 10^6^ cells/mL. For apoptosis analysis, the cell suspension (300 *μ*L) was incubated with 5 *μ*L Annexin V and 3 *μ*L propidium iodide in the dark for 10–15 min at room temperature, the apoptotic cells were quantified using a FACSort Flow Cytometer (BD, San Jose, CA). For cell cycle analysis, the cells were collected and washed with ice‐cold PBS for two times. After centrifuge, the cells were fixed in 70% ethanol overnight at 4°C. The cells were suspended in the staining solution containing 50 *μ*g/mL propidium iodide and 0.1% ribonuclease A (RNase A) for 30 min at room temperature. Cell cycle was analyzed by Flow Cytometry.

### *In vivo* tumor growth

The *in vivo* animal study was approved by the Animal Ethics Committee of Central South University. HCC827 cells (1 × 10^6^/100 *μ*L) was counted and suspended in RPMI‐1640 medium, then the cell suspension was inoculated s.c. into the right flank of 5‐week‐old female athymic nude mice. When average tumor volume reached around 50 mm^3^, an i.p. injection of oxymatrine at a dose of 50 mg/kg was initiated and repeated every 3 days. The control group was administered vehicle. The tumor volume was recorded by Vernier caliper and calculated following the formula of A × B^2^ × 0.5. A is the longest diameter of tumor, B is the shortest diameter and B^2^ is B squared.

### Immunohistochemical analysis of tumor tissue

Immunohistochemical (IHC) staining was performed as described previously 23. Briefly, the slide was baked at 60°C for 2 h. After deparaffinization and rehydration, the slide was submerged into boiling sodium citrate buffer (10 mmol/L, pH 6.0) for 10 min, and incubated with 3% H_2_O_2_ for 10 min at room temperature. 50% goat serum albumin in 1 × PBS was used for blocking. Then the slide was incubated with a primary antibody in a humidified chamber at 4°C overnight. The slide was washed with ice‐cold PBS and hybridized with the secondary antibody for 1 h at room temperature. Hematoxylin was used for counterstaining. The intensity was viewed by Image‐Pro PLUS (v.6) software programs.

### Statistical analysis

The quantitative data were expressed as mean values ± SD of at least three independent experiments. Significant differences were determined by Student's *t‐*test, *P *< 0.05 was considered statistical significance.

## Results

### Oxymatrine inhibits NSCLC cell growth

**Figure 1:**
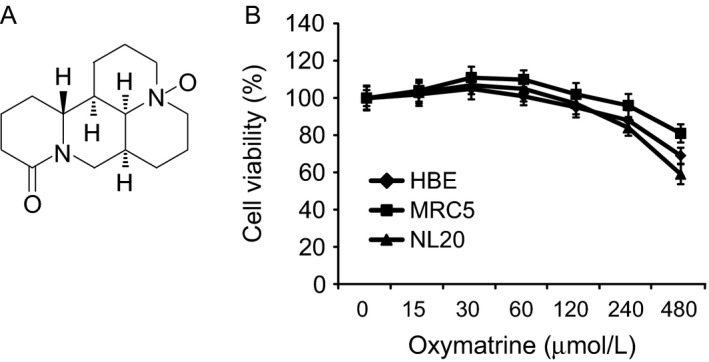
The structure and cytotoxicity of oxymatrine. (A) The chemical structure of oxymatrine. (B) Cytotoxicity of oxymatrine was measured in normal lung cells by MTS assay. MRC‐5, NL20, and HBE cells were treated with various concentrations of oxymatrine for 24 h. Data are shown as means ± SD from triplicate experiments.

**Figure 2:**
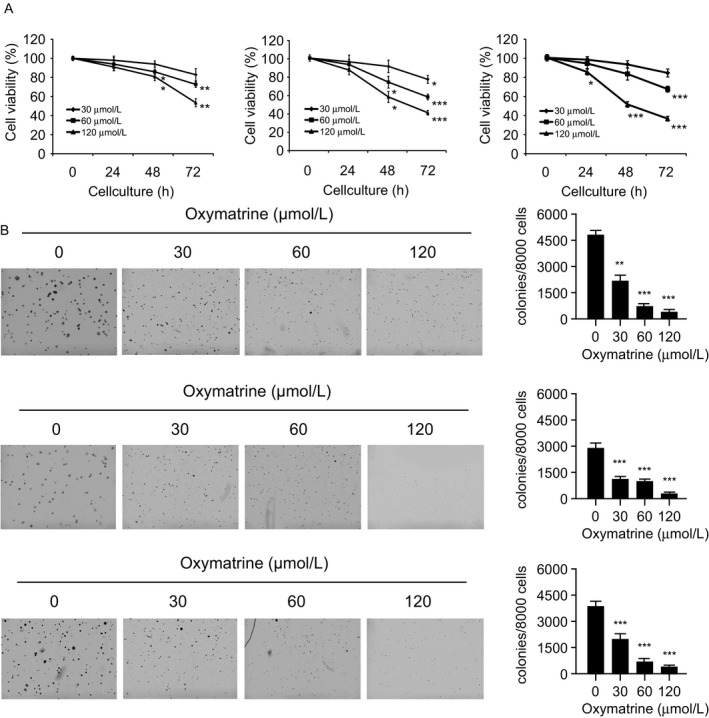
Inhibitory effects of oxymatrine on NSCLC cells. (A) Oxymatrine inhibits anchorage‐dependent cell growth in a panel of human NSCLC cells, including A549 (left), H1975 (middle), and HCC827 (right). Cell viability was measured by MTS assay. (B) Three NSCLC cell lines, including A549 (upper), H1975 (middle), and HCC827 (bottom) were subjected to the soft agar assay. The average colony number was calculated from three separate experiments. Asterisk, significant (*P < 0.05, **P < 0.01, ***P < 0.001) suppression of cell viability or colony formation by oxymatrine compared with the DMSO‐treated group.

Oxymatrine (Fig. 1A, MW. 264.36) has shown antitumor activities against several types of human cancers. In order to identify the antitumor effect of oxymatrine on NSCLC, we first investigated whether oxymatrine exerts any cytotoxic effect on normal human lung cells. HBE, MRC‐5, and NL20 cells were treated with oxymatrine, and cell viability was measured by MTS assay. The result showed that oxymatrine had no obvious inhibitory effect against these normal lung cells at concentrations ≤ 240 *μ*mol/L (Fig. 1B). We next detected the suppression effect of oxymatrine on cell proliferation in A549, H1975, and HCC827cells. MTS results indicated that higher concentration (≥60 *μ*mol/L), or long‐term (≥48 h) treatment with oxymatrine dramatically suppressed cell proliferation (Fig. 2A). We then examined the inhibitory efficiency of oxymatrine on anchorage‐independent growth of these NSCLC cells. As we expected, oxymatrine could potently inhibit the colony formation of NSCLC cells even at 30 *μ*mol/L. More importantly, oxymatrine almost blocked the anchorage‐independent growth of these three NSCLC cells when the concentration reached at 120 *μ*mol/L (Fig. 2B). These results suggest that oxymatrine specifically suppresses the growth of NSCLC cells, but has no obvious cytotoxic effect on normal lung cells.

### Oxymatrine suppresses the EGFR signaling pathway

**Figure 3:**
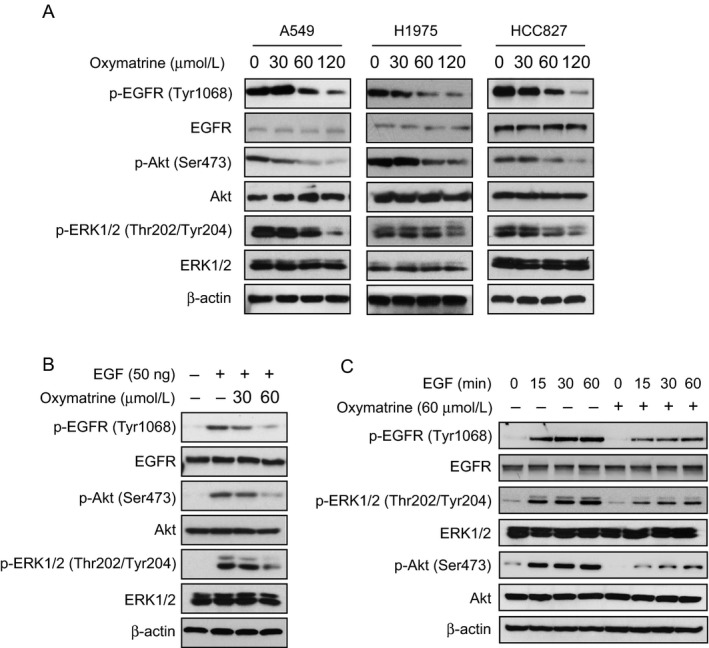
Oxymatrine suppresses EGFR signaling pathway. (A) Oxymatrine inhibits the activity of EGFR signaling pathway. A549 (left), H1975 (middle), and HCC827 (right) cells were treated with oxymatrine for 24 h as indicated, Western blot was conducted to detect target proteins. (B) Oxymatrine downregulates EGF‐induced EGFR signaling pathway activation. HCC827 cells were starved overnight and then treated with oxymatrine at the indicated concentrations for 2 h. After stimulation with EGF (0, 50 ng/mL) for 30 min, the cells were harvested and protein levels were determined by Western blotting. (C) Oxymatrine downregulates EGF‐induced EGFR signaling pathway activation. HCC827 cells were starved overnight and then treated with 60 *μ*mol/L oxymatrine for 2 h. After stimulation with EGF (50 ng/mL) for various time points, the cells were harvested and protein levels were determined by Western blotting.

EGFR signaling pathway plays a crucial role in lung tumorigenesis. We then determined whether oxymatrine had any effect on EGFR activation. We chose three NSCLC cell lines, including the A549, H1975, and HCC827 cells, which harbor the WT EGFR, L858R/T790M, and Exon 19 deletion mutations, respectively. Immunoblotting analysis indicated that the phosphorylation of EGFR was decreased in response to oxymatrine treatment in these three cell lines, which suggested that oxymatrine can effectively inhibit the activation of both WT and activating mutations of EGFR (Fig. 3A). Moreover, we also found that the activation of EGFR downstream target genes, such as Akt and ERK1/2, were substantially suppressed after oxymatrine treatment (Fig. 3A). Further study showed that oxymatrine treatment strikingly inhibited EGF‐induced EGFR, Akt, and ERK1/2 activation in HCC827 cells (Fig. 3B and C). These results suggest that oxymatrine suppresses the EGFR signaling pathway.

### Oxymatrine induces G0/G1 cell cycle arrest and decreases cyclin D1

**Figure 4:**
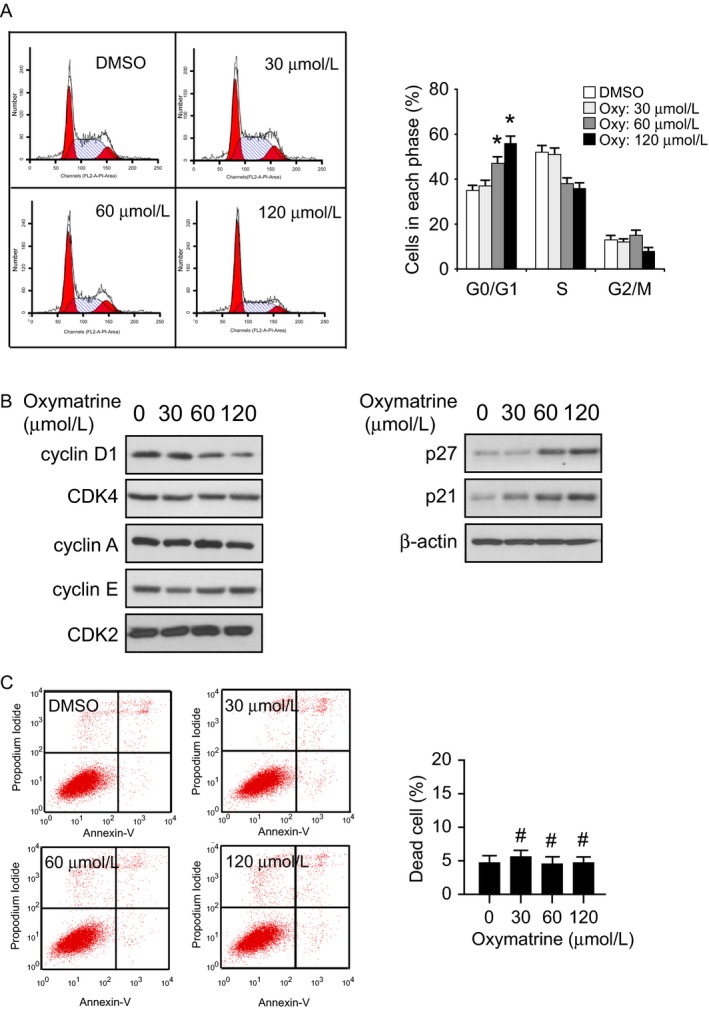
Oxymatrine induces G0/G1 cell cycle arrest in HCC827 cells. (A) HCC827 cells were treated with various concentrations of oxymatrine for 24 h as indicated, flow cytometry was conducted to analyze cell cycle distribution (*P < 0.05 vs. DMSO‐treated). (B) HCC827 cells were treated with various concentrations of oxymatrine for 24 h as indicated, Western blot was conducted to detect target proteins. (C) HCC827 cells were treated with various concentrations of oxymatrine for 24 h as indicated, the percentage of apoptosis cells was quantified by flow cytometry (#, not statistically significant).

We next tested the effect of oxymatrine on cell cycle progression in HCC827 cells. After exposure to 60 or 120 *μ*mol/L oxymatrine for 24 h, the cell proportion of G0/G1 phase were reached around 50% or 60% (Fig. 4A) in HCC827 cells. Additionally, the cells in S phase were decreased from 50% to 40%. This result suggested the inhibition or delay in DNA replication/synthesis and cell proliferation. The Western blotting data showed that oxymatrine dramatically decreased the expression of cyclin D1, but did not downregulate the protein levels of cyclin A and cyclin E. Moreover, the CDK inhibitors, including p21 and p27, were increased in oxymatrine‐treated groups (Fig. 4B). The Flow Cytometry results showed that oxymatrine did not markedly induce apoptosis even at the relatively higher concentration of 120 *μ*mol/L (Fig. 4C). These results indicate that oxymatrine‐mediated HCC827 cell growth inhibition may partly dependent on G0/G1 cell cycle arrest.

### Akt inhibition is required for oxymatrine‐mediated cell cycle arrest in HCC827 cells

**Figure 5:**
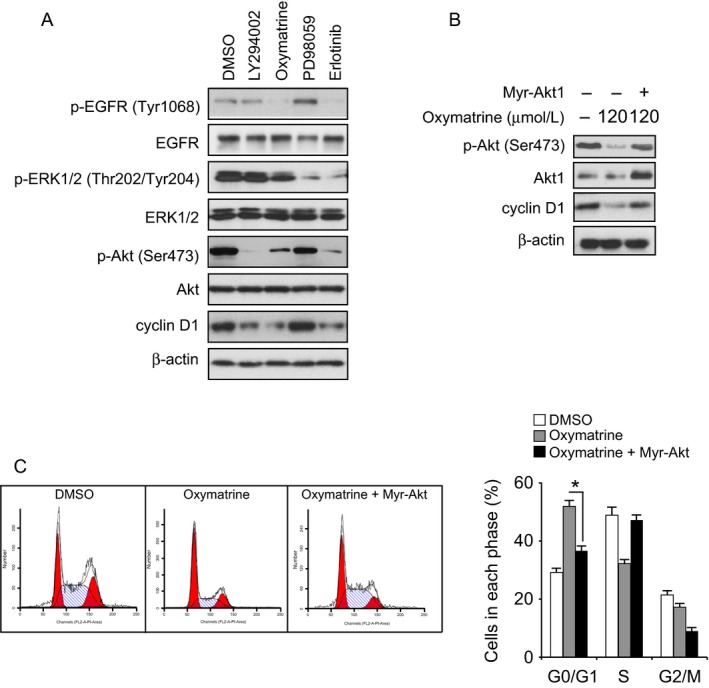
Akt inhibition is required for oxymatrine‐mediated cell cycle arrest. (A) HCC827 cells were treated with oxymatrine or inhibitors for 24 h as indicated, protein levels were determined by Western blotting. (B) Overexpression of constitutively activated Akt (Myr‐Akt1) rescues cyclin D1 expression. The Myr‐Akt1 plasmid was transfected into HCC827 cells, after 24 h, these cells were treated with oxymatrine for another 24 h as indicated. Western blot analysis was performed to detect the protein expression levels. (C) Overexpression of Myr‐Akt1 rescues cell cycle arrest in oxymatrine‐treated HCC827 cells. The Myr‐Akt1 plasmid transfection and oxymatrine treatment were performed as indicated. Cell cycle distribution was analyzed by flow cytometry (*P < 0.05 vs. oxymatrine‐treated).

Our results demonstrated that oxymatrine inhibited human NSCLC cells growth and EGFR signaling pathway activation. In order to further determine that oxymatrine‐induced inhibition of cell cycle progression is dependent on the suppression of EGFR signaling pathway, we selectively inhibited EGFR, Akt, and ERK1/2 kinase activity via small‐molecule inhibitors in HCC827 cells. Our results indicated that suppression of EGFR activation by oxymatrine and EGFR inhibitor, erlotinib, resulted in downregulation of cyclin D expression. Furthermore, the Akt inhibitor, LY294002, dramatically decreased cyclin D1 expression in HCC827 cells (Fig. 5A). However, downregulation of ERK1/2 phosphorylation had no obvious effect on cyclin D1 expression (Fig. 5A). We further found that overexpression of constitutively activated Akt1 (myr‐Akt1) rescued the cyclin D1 expression in oxymatrine‐treated HCC827 cells (Fig. 5B). Additionally, the flow cytometry data indicated that myr‐Akt1 transfection rescued oxymatrine‐induced cell cycle arrest in HCC827 cells, overexpression of myr‐Akt1 increased the cell proportion of S phase (Fig. 5C). Our data suggest that oxymatrine‐regulated G0/G1 cell cycle arrest is partly dependent on Akt activation modulation.

### Oxymatrine inhibits *in vivo* tumor growth

**Figure 6:**
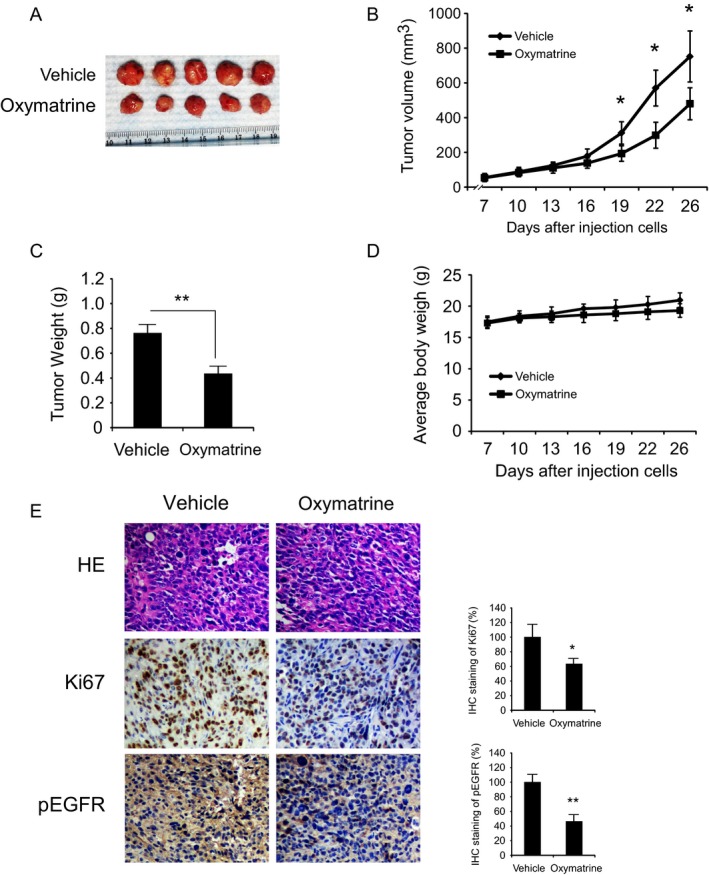
Oxymatrine inhibits tumor growth in HCC827 xenograft mouse model. (A) HCC827 cells were subcutaneously injected into the right flank of mice. At the treatment endpoint, mice were killed and tumors were removed, weighed, and photographed. (B) Tumor volumes were measured twice a week. (C) The tumor weight from the vehicle‐ and oxymatrine‐treated group was measured. (D) During the treatment period, the body weight of mice was measured twice a week to determine the effect of oxymatrine. For (B), (C), and (D), data are shown as mean values ± SD obtained from five mice in each group. (E) Immunohistochemical staining examination of Ki67 and p‐EGFR in tumor sections from the vehicle‐ or the oxymatrine‐treated group. The integrated optical density (IOD) was evaluated using the Image‐Pro Plus software (version 6.2) program. (*P < 0.05, **P < 0.01 vs. vehicle).

We further determined the antitumor effects of oxymatrine on NSCLC cells in a xenograft mouse model. HCC827 cells were transplanted into the right flank of 6‐week‐old female athymic nude mice. Oxymatrine (50 mg/kg per day) or vehicle treatment was initiated when the average tumor volume reached ≥50 mm^3^. Results indicated that the final average tumor volume of the vehicle‐treated group was around 752.02 ± 146.76 mm^3^, whereas average tumor size of the oxymatrine‐treated group was 479.92 ± 91.89 mm^3^ (Fig. 6A and B). The average tumor weights of the vehicle‐treated group and oxymatrine‐treated group were 0.77 ± 0.08 g and 0.47 ± 0.05 g, respectively (Fig. 6C). During the treatment period, oxymatrine did not affect body weight of the mice (Fig. 6D). IHC analysis showed that oxymatrine substantially inhibited the phosphorylation of EGFR in HCC827 xenograft tumors. Moreover, the protein level of Ki67 was decreased in oxymatrine‐treated group (Fig. 6E). Our results indicate that oxymatrine inhibits tumor growth *in vivo*.

## Discussion

Increased levels of EGFR gene expression are observed in human cancers. Previous studies have reported that EGFR was overexpressed in 62% of NSCLC cases, and its high expression is correlated with a poor prognosis 24, 25. More importantly, EGFR mutations have been identified in approximately 10–30% of NSCLC 26. The most common mutations are in‐frame deletions of exon 19 and the L858R point mutation in exon 21, which result in hyperactivation of EGFR signaling pathway 27. Since EGFR plays a crucial role in NSCLC, the EGFR‐targeted therapeutics currently represents the best‐studied example of oncogene addiction in human NSCLC. The tyrosine kinase inhibitors (EGFR‐TKIs) of EGFR was designed, and this kind of small‐molecule compounds reversibly or irreversibly bind to the ATP binding pocket of the mutated EGFR, thereby suppressing the phosphorylation of the EGFR signaling 28, 29, 30.

Although the clinical application of tyrosine kinase inhibitors, including gefitinib and erlotinib, have shown a dramatic prolong survival in NSCLC patients with EGFR activating mutations, most patients eventually develop acquired resistance 31. The emergence of a secondary mutation, T790M, accounts for one‐half of acquired resistance to TKIs, whereas approximately 20% of the acquired resistance cases are associated with the activation of ErbB3/PI3K/Akt signaling mediated by c‐Met amplification 27, 32. Thus, the second‐generation TKIs, such as afatinib, and the ^3^rd generation TKIs, including osimertinib, have been developed 7, 33, 34. However, the TKI resistance in human NSCLC has increased clinically. More importantly, only the activating mutation harbored patients respond to TKI treatment 35. Thus, further discover novel therapeutic targets or develop new chemicals are still an urgent demand for clinical NSCLC treatment.

In this study, we reported firstly that the natural compound, oxymatrine, exerts an antitumor effect on NSCLC via directly inhibits the EGFR signaling (Fig. 3). The calculated IC50 (72 h) value of oxymatrine on A549, H1975, and HCC827 cells, which harbors the WT EGFR, L858R/T790M, and Exon 19 deletion mutated EGFR, was around 145 *μ*mol/L, 98 *μ*mol/L, and 82 *μ*mol/L, respectively (data not shown). Our results indicated that oxymatrine is much more inhibition effective on Exon 19 deletion mutated EGFR and L858R/T790M mutated EGFR than WT EGFR. The MTS results demonstrated that oxymatrine had no obvious cytotoxicity on normal lung cells at concentrations ≤ 240 *μ*mol/L, but markedly inhibited NSCLC at concentrations ≥ 60 *μ*mol/L. Importantly, the *in vivo* data showed that the consumption of oxymatrine did not induce significant body weight loss occurred in the oxymatrine‐treated group (Fig. 6). These results suggested that oxymatrine inhibited NSCLC via targeting EGFR signaling but has no obvious cytotoxicity on normal cells. Recently, Liu et al. found that oxymatrine synergistically enhances the antitumor activity of oxaliplatin in colon carcinoma 36 and enhances the inhibitory effect of 5‐fluorouracil on hepatocellular carcinoma *in vitro* and *in vivo* 15. Thus, oxymatrine owns the potential to serve as a sensitizing agent for NSCLC treatment via combination with other drugs.

Deregulation of the cell cycle is one of the hallmarks of human cancer 37, 38. Cyclin D1 plays a crucial role in the regulation of the cell cycle G1‐S transition, and compelling evidences have demonstrated that cyclin D1 is frequently amplified and overexpressed in human NSCLS 39, 40. Alterations of the pathways regulated by growth factors such as EGF, and by the *ras* oncogene product may contribute to cyclin D1 expression 40. Evidence from laboratory investigation discovered that inhibition of EGFR activity by TKIs dramatically suppressed the expression of cyclin D1 protein 41, 42, 43 in NSCLC. Here, we found that oxymatrine‐mediated cyclin D1 downregulation was dependent on the suppression of EGFR‐Akt signaling, exogenous overexpression of Myr‐Akt rescued cyclin D1 expression in the oxymatrine‐treated group (Figs. 4 and 5). However, inhibition of ERK1/2 had no obvious effect on cyclin D1 expression (Fig. 5A). Moreover, recent studies indicated that EGFR can translocate to the nucleus and act as a transcription factor or kinase in human cancers 44, 45, 46. The anticancer treatment, such as radiation and EGFR‐targeted therapy, or other stimuli, including ligand binding, substantially induced EGFR nuclear localization 46, 47. The nuclear EGFR regulates gene expression, such as promotes cyclin D1 transcription 48, 49. Although our results showed that oxymatrine‐induced cyclin D1 downregulation was partly dependent on EGFR‐Akt kinases activity, there is still a possibility that oxymatrine directly inhibited EGFR nuclear translocation and EGFR‐mediated cyclin D1 transcription regulation.

Overall, our data implied that suppression of EGFR signaling pathway is involved in oxymatrine‐induced tumor inhibition in NSCLC. We analyzed the suppression effect of oxymatrine against WT EGFR, exon 19 deletion and the L858R/T790M mutated EGFR *in vitro*. For the first time, we identified that decreases the activity of the EGFR‐Akt‐cyclin D1 signaling pathway was one of the major underlying mechanisms for oxymatrine‐induced cell cycle arrest in human NSCLC.

## Conflicts of Interest

No potential conflicts of interest were disclosed.

## References

[1] Siegel, R. , D. Naishadham , and A. Jemal . 2013 Cancer statistics, 2013. CA Cancer J. Clin. 63:11–30. 23335087 10.3322/caac.21166

[2] DeSantis, C. E. , C. C. Lin , A. B. Mariotto , R. L. Siegel , K. D. Stein , J. L. Kramer , et al. 2014 Cancer treatment and survivorship statistics, 2014. CA Cancer J. Clin. 64:252–271. 24890451 10.3322/caac.21235

[3] Thomas, A. , S. V. Liu , D. S. Subramaniam , and G. Giaccone . 2015 Refining the treatment of NSCLC according to histological and molecular subtypes. Nat. Rev. Clin. Oncol. 12:511–526. 25963091 10.1038/nrclinonc.2015.90

[4] Rosell, R. , and N. Karachaliou . 2015 Lung cancer in 2014: optimizing lung cancer treatment approaches. Nat. Rev. Clin. Oncol. 12:75–76. 25533943 10.1038/nrclinonc.2014.225

[5] Herbst, R. S. , J. V. Heymach , and S. M. Lippman . 2008 Lung cancer. N. Engl. J. Med. 359:1367–1380. 18815398 10.1056/NEJMra0802714 PMC10662965

[6] Wang, Y. , M. Wang , Q. Wang , Z. Geng , and M. Sun . 2017 Incidence and risk of infections associated with EGFR‐TKIs in advanced non‐small‐cell lung cancer: a systematic review and meta‐analysis of randomized controlled trials. Oncotarget 8:29406–29415. 28107192 10.18632/oncotarget.14707 PMC5438740

[7] Liao, B. C. , C. C. Lin , J. H. Lee , and J. C. Yang . 2017 Optimal management of EGFR‐mutant non‐small cell lung cancer with disease progression on first‐line tyrosine kinase inhibitor therapy. Lung Cancer 110:7–13. 28676222 10.1016/j.lungcan.2017.05.009

[8] Liu, T. C. , X. Jin , Y. Wang , and K. Wang . 2017 Role of epidermal growth factor receptor in lung cancer and targeted therapies. Am. J. Cancer Res. 7:187–202. 28337370 PMC5336495

[9] Castellanos, E. , E. Feld , and L. Horn . 2017 Driven by mutations: the predictive value of mutation subtype in EGFR‐mutated non‐small cell lung cancer. J. Thorac. Oncol. 12:612–623. 28017789 10.1016/j.jtho.2016.12.014

[10] Normanno, N. , M. R. Maiello , N. Chicchinelli , A. Iannaccone , C. Esposito , R. De Cecio , et al. 2017 Targeting the EGFR T790M mutation in non‐small‐cell lung cancer. Expert Opin. Ther. Targets 21:159–165. 28002980 10.1080/14728222.2017.1272582

[11] Liu, Y. , Y. Xu , W. Ji , X. Li , B. Sun , Q. Gao , et al. 2014 Anti‐tumor activities of matrine and oxymatrine: literature review. Tumour Biol. 35:5111–5119. 24526416 10.1007/s13277-014-1680-z

[12] Lin, B. , D. Li , and L. Zhang . 2016 Oxymatrine mediates Bax and Bcl‐2 expression in human breast cancer MCF‐7 cells. Pharmazie 71:154–157. 27183711

[13] Guo, B. , T. Zhang , J. Su , K. Wang , and X. Li . 2015 Oxymatrine targets EGFR(p‐Tyr845) and inhibits EGFR‐related signaling pathways to suppress the proliferation and invasion of gastric cancer cells. Cancer Chemother. Pharmacol. 75:353–363. 25527205 10.1007/s00280-014-2651-1

[14] Li, S. , Y. Zhang , Q. Liu , Q. Zhao , L. Xu , S. Huang , et al. 2017 Oxymatrine inhibits proliferation of human bladder cancer T24 cells by inducing apoptosis and cell cycle arrest. Oncol. Lett. 13:4453–4458. 28588714 10.3892/ol.2017.6013 PMC5452914

[15] Liu, Y. , T. Bi , W. Dai , G. Wang , L. Qian , Q. Gao , et al. 2016 Oxymatrine synergistically enhances the inhibitory effect of 5‐fluorouracil on hepatocellular carcinoma *in vitro* and *in vivo* . Tumour Biol. 37:7589–7597. 26687645 10.1007/s13277-015-4642-1

[16] Wang, X. , C. Liu , J. Wang , Y. Fan , Z. Wang , and Y. Wang . 2017 Oxymatrine inhibits the migration of human colorectal carcinoma RKO cells via inhibition of PAI‐1 and the TGF‐beta1/Smad signaling pathway. Oncol. Rep. 37:747–753. 27959430 10.3892/or.2016.5292 PMC5355745

[17] Li, J. , K. Jiang , and F. Zhao . 2015 Oxymatrine suppresses proliferation and facilitates apoptosis of human ovarian cancer cells through upregulating microRNA29b and downregulating matrix metalloproteinase2 expression. Mol. Med. Rep. 12:5369–5374. 26099492 10.3892/mmr.2015.3977

[18] Wu, C. , W. Huang , Y. Guo , P. Xia , X. Sun , X. Pan , et al. 2015 Oxymatrine inhibits the proliferation of prostate cancer cells *in vitro* and *in vivo* . Mol. Med. Rep. 11:4129–4134. 25672672 10.3892/mmr.2015.3338 PMC4394963

[19] He, M. , L. Jiang , B. Li , G. Wang , J. Wang , and Y. Fu . 2017 Oxymatrine suppresses the growth and invasion of MG63 cells by up‐regulating PTEN and promoting its nuclear translocation. Oncotarget 8:65100–65110. 29029415 10.18632/oncotarget.17783 PMC5630315

[20] Chen, H. , J. Zhang , J. Luo , F. Lai , Z. Wang , H. Tong , et al. 2013 Antiangiogenic effects of oxymatrine on pancreatic cancer by inhibition of the NF‐kappaB‐mediated VEGF signaling pathway. Oncol. Rep. 30:589–595. 23754270 10.3892/or.2013.2529

[21] Li, W. , C. Peng , M. H. Lee , D. Lim , F. Zhu , Y. Fu , et al. 2013 TRAF4 is a critical molecule for Akt activation in lung cancer. Cancer Res. 73:6938–6950. 24154876 10.1158/0008-5472.CAN-13-0913 PMC3856436

[22] Yu, X. , W. Li , Q. Deng , S. You , H. Liu , S. Peng , et al. 2017 Neoalbaconol inhibits angiogenesis and tumor growth by suppressing EGFR‐mediated VEGF production. Mol. Carcinog. 56:1414–1426. 27996164 10.1002/mc.22602

[23] Liu, H. , W. Li , X. Yu , F. Gao , Z. Duan , X. Ma , et al. 2016 EZH2‐mediated Puma gene repression regulates non‐small cell lung cancer cell proliferation and cisplatin‐induced apoptosis. Oncotarget 7:56338–56354. 27472460 10.18632/oncotarget.10841 PMC5302918

[24] Nicholson, R. I. , J. M. Gee , and M. E. Harper . 2001 EGFR and cancer prognosis. Eur. J. Cancer 37(Suppl. 4):S9–S15. 11597399 10.1016/s0959-8049(01)00231-3

[25] Krause, D. S. , and R. A. Van Etten . 2005 Tyrosine kinases as targets for cancer therapy. N. Engl. J. Med. 353:172–187. 16014887 10.1056/NEJMra044389

[26] Cancer Genome Atlas Research . 2014 N. Comprehensive molecular profiling of lung adenocarcinoma. Nature 511:543–550. 25079552 10.1038/nature13385 PMC4231481

[27] Sharma, S. V. , D. W. Bell , J. Settleman , and D. A. Haber . 2007 Epidermal growth factor receptor mutations in lung cancer. Nat. Rev. Cancer 7:169–181. 17318210 10.1038/nrc2088

[28] Herbst, R. S. , M. Fukuoka , and J. Baselga . 2004 Gefitinib–a novel targeted approach to treating cancer. Nat. Rev. Cancer 4:956–965. 15573117 10.1038/nrc1506

[29] Lamb, Y. N. , and L. J. Scott . 2017 Osimertinib: a review in T790M‐positive advanced non‐small cell lung cancer. Target Oncol. 12:555–562. 28710746 10.1007/s11523-017-0519-0

[30] Li, X. , and C. Zhou . 2017 Comparison of cross‐platform technologies for EGFR T790M testing in patients with non‐small cell lung cancer. Oncotarget. 2017 Jul 5. https://doi.org/10.18632/oncotarget.19007. 10.18632/oncotarget.19007 PMC5725066 29246024

[31] Jackman, D. , W. Pao , G. J. Riely , J. A. Engelman , M. G. Kris , P. A. Janne , et al. 2010 Clinical definition of acquired resistance to epidermal growth factor receptor tyrosine kinase inhibitors in non‐small‐cell lung cancer. J. Clin. Oncol. 28:357–360. 19949011 10.1200/JCO.2009.24.7049 PMC3870288

[32] Engelman, J. A. , K. Zejnullahu , T. Mitsudomi , Y. Song , C. Hyland , J. O. Park , et al. 2007 MET amplification leads to gefitinib resistance in lung cancer by activating ERBB3 signaling. Science 316:1039–1043. 17463250 10.1126/science.1141478

[33] Proto, C. , G. Lo Russo , G. Corrao , M. Ganzinelli , F. Facchinetti , R. Minari , et al. 2017 Treatment in EGFR‐mutated non‐small cell lung cancer: how to block the receptor and overcome resistance mechanisms. Tumori 103:325–337. 28708233 10.5301/tj.5000663

[34] Ricciuti, B. , S. Baglivo , L. Paglialunga , A. De Giglio , G. Bellezza , R. Chiari , et al. 2017 Osimertinib in patients with advanced epidermal growth factor receptor T790M mutation‐positive non‐small cell lung cancer: rationale, evidence and place in therapy. Ther. Adv. Med. Oncol. 9:387–404. 28607578 10.1177/1758834017702820 PMC5455880

[35] Paez, J. G. , P. A. Janne , J. C. Lee , S. Tracy , H. Greulich , S. Gabriel , et al. 2004 EGFR mutations in lung cancer: correlation with clinical response to gefitinib therapy. Science 304:1497–1500. 15118125 10.1126/science.1099314

[36] Liu, Y. , T. Bi , Z. Wang , G. Wu , L. Qian , Q. Gao , et al. 2016 Oxymatrine synergistically enhances antitumor activity of oxaliplatin in colon carcinoma through PI3K/AKT/mTOR pathway. Apoptosis 21:1398–1407. 27671687 10.1007/s10495-016-1297-3

[37] Otto, T. , and P. Sicinski . 2017 Cell cycle proteins as promising targets in cancer therapy. Nat. Rev. Cancer 17:93–115. 28127048 10.1038/nrc.2016.138 PMC5345933

[38] Hanahan, D. , and R. A. Weinberg . 2011 Hallmarks of cancer: the next generation. Cell 144:646–674. 21376230 10.1016/j.cell.2011.02.013

[39] Gautschi, O. , D. Ratschiller , M. Gugger , D. C. Betticher , and J. Heighway . 2007 Cyclin D1 in non‐small cell lung cancer: a key driver of malignant transformation. Lung Cancer 55:1–14. 17070615 10.1016/j.lungcan.2006.09.024

[40] Musgrove, E. A. , C. E. Caldon , J. Barraclough , A. Stone , and R. L. Sutherland . 2011 Cyclin D as a therapeutic target in cancer. Nat. Rev. Cancer 11:558–572. 21734724 10.1038/nrc3090

[41] Petty, W. J. , K. H. Dragnev , V. A. Memoli , Y. Ma , N. B. Desai , A. Biddle , et al. 2004 Epidermal growth factor receptor tyrosine kinase inhibition represses cyclin D1 in aerodigestivetract cancers. Clin. Cancer Res. 10:7547–7554. 15569985 10.1158/1078-0432.CCR-04-1169

[42] Kalish, L. H. , R. A. Kwong , I. E. Cole , R. M. Gallagher , R. L. Sutherland , and E. A. Musgrove . 2004 Deregulated cyclin D1 expression is associated with decreased efficacy of the selective epidermal growth factor receptor tyrosine kinase inhibitor gefitinib in head and neck squamous cell carcinoma cell lines. Clin. Cancer Res. 10:7764–7774. 15570011 10.1158/1078-0432.CCR-04-0012

[43] Kobayashi, S. , T. Shimamura , S. Monti , U. Steidl , C. J. Hetherington , A. M. Lowell , et al. 2006 Transcriptional profiling identifies cyclin D1 as a critical downstream effector of mutant epidermal growth factor receptor signaling. Cancer Res. 66:11389–11398. 17145885 10.1158/0008-5472.CAN-06-2318

[44] Li, H. , L. You , J. Xie , H. Pan , and W. Han . 2017 The roles of subcellularly located EGFR in autophagy. Cell. Signal. 35:223–230. 28428083 10.1016/j.cellsig.2017.04.012

[45] Brand, T. M. , M. Iida , N. Luthar , M. M. Starr , E. J. Huppert , and D. L. Wheeler . 2013 Nuclear EGFR as a molecular target in cancer. Radiother. Oncol. 108:370–377. 23830194 10.1016/j.radonc.2013.06.010 PMC3818450

[46] Dittmann, K. , C. Mayer , and H. P. Rodemann . 2010 Nuclear EGFR as novel therapeutic target: insights into nuclear translocation and function. Strahlenther. Onkol. 186:1–6. 20082181 10.1007/s00066-009-2026-4

[47] Han, W. , and H. W. Lo . 2012 Landscape of EGFR signaling network in human cancers: biology and therapeutic response in relation to receptor subcellular locations. Cancer Lett. 318:124–134. 22261334 10.1016/j.canlet.2012.01.011 PMC3304012

[48] Lin, S. Y. , K. Makino , W. Xia , A. Matin , Y. Wen , K. Y. Kwong , et al. 2001 Nuclear localization of EGF receptor and its potential new role as a transcription factor. Nat. Cell Biol. 3:802–808. 11533659 10.1038/ncb0901-802

[49] Bazzani, L. , S. Donnini , F. Finetti , G. Christofori , and M. Ziche . 2017 PGE2/EP3/SRC signaling induces EGFR nuclear translocation and growth through EGFR ligands release in lung adenocarcinoma cells. Oncotarget 8:31270–31287. 28415726 10.18632/oncotarget.16116 PMC5458206

